# Effects of a leaf spring structured midsole on joint mechanics and lower limb muscle forces in running

**DOI:** 10.1371/journal.pone.0172287

**Published:** 2017-02-24

**Authors:** Tobias Wunsch, Nathalie Alexander, Josef Kröll, Thomas Stöggl, Hermann Schwameder

**Affiliations:** Department of Sport Science and Kinesiology, University of Salzburg, Salzburg, Austria; Northwestern University, UNITED STATES

## Abstract

To enhance running performance in heel-toe running, a leaf spring structured midsole shoe (LEAF) has recently been introduced. The purpose of this study was to investigate the effect of a LEAF compared to a standard foam midsole shoe (FOAM) on joint mechanics and lower limb muscle forces in overground running. Nine male long-distance heel strike runners ran on an indoor track at 3.0 ± 0.2 m/s with LEAF and FOAM shoes. Running kinematics and kinetics were recorded during the stance phase. Absorbed and generated energy (negative and positive work) of the hip, knee and ankle joint as well as muscle forces of selected lower limb muscles were determined using a musculoskeletal model. A significant reduction in energy absorption at the hip joint as well as energy generation at the ankle joint was found for LEAF compared to FOAM. The mean lower limb muscle forces of the m. soleus, m. gastrocnemius lateralis and m. gastrocnemius medialis were significantly reduced for LEAF compared to FOAM. Furthermore, m. biceps femoris showed a trend of reduction in running with LEAF. The remaining lower limb muscles analyzed (m. gluteus maximus, m. rectus femoris, m. vastus medialis, m. vastus lateralis, m. tibialis anterior) did not reveal significant differences between the shoe conditions. The findings of this study indicate that LEAF positively influenced the energy balance in running by reducing lower limb muscle forces compared to FOAM. In this way, LEAF could contribute to an overall increased running performance in heel-toe running.

## Introduction

From a biomechanical perspective, the mechanical energy generated by the muscles of the lower limb joints enables the runner to fulfil the movement task and determines running performance. In the literature, three major strategies have been proposed to improve the mechanical energy cost in running to enhance running performance: (1) storage and return of energy (2) optimization of the muscle functions by enabling muscles to work at an optimal force-velocity and force-length relationship, and (3) minimization of energy loss [1, 2].

Running shoe designs have had limited success in applying the concept of energy return [1, 3] and the concept of functional optimization of the musculoskeletal system lacks scientific support [4]. Therefore, Nigg and Segesser [2] suggested that running shoe designs should focus on strategies to minimize energy loss. Consequently, material [5, 6] and structural changes of running shoe midsoles have been considered [7, 8].

**Fig 1:**
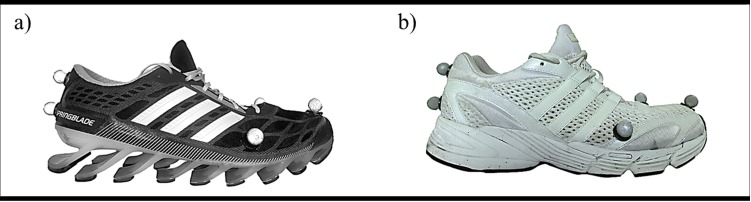
Midsole designs. (a) Leaf spring structured midsole shoe (LEAF). (b) Standard foam midsole shoe (FOAM). The shoes are shown with reflective markers attached.

One of these shoe designs is a leaf spring structured midsole shoe (LEAF, Fig 1A). In contrast to a standard foam midsole shoe (FOAM, Fig 1B) the midsole of LEAF consists of a series of non-linked leaf springs. The LEAF shoe uses the midsole deformation induced by the vertical ground reaction force for shifting the shoe anteriorly during the first part of stance phase in heel-toe running [7]. When using LEAF compared to FOAM in treadmill running, the anterior foot shift resulted in increased stride length and improved running economy [7].

Based on these findings, one could presume a positive effect of LEAF compared to FOAM on the mechanical energy cost of the human locomotor system. However, it is important to determine the sources and magnitudes of energy absorption and generation to achieve a deeper understanding of the potential mechanisms on how this specific footwear affects the overall energy cost in running [9]. Independent of the footwear joint power analysis during stance phase has indicated that in each joint energy is either absorbed (time-integrated negative power) or generated (time-integrated positive power) in distinct phases [9]. It has been shown that LEAF leads to an anterior foot shift during the first part of stance [7], therefore, it can be hypothesized that LEAF compared to FOAM affects especially the first part of stance showing reduced energy absorption of the lower limb joints.

While the absorption of energy is considered to be eccentric muscle activity, the generation of energy is related to concentric muscle activity [9]. To determine the forces produced by the lower limb muscles during running, inverse dynamic musculoskeletal models can be used [10–13]. These models are constrained to estimate the raw muscle forces from the previously calculated joint moments [14]. In this way musculoskeletal models can help to identify those single muscles differing in running with LEAF compared to FOAM [9]. In case that LEAF affects the energy contribution of hip, knee and ankle joint it seems plausible that also lower limb muscle forces are affected by the midsole design. Because muscles consume metabolic energy [15], these differences in muscle forces could be relevant for explaining differences in running economy between LEAF and FOAM [7]. Within lower limb muscles the m. biceps femoris was identified to have the greatest impact on running economy [16].

Based on these considerations it was hypothesized that running with LEAF compared to FOAM shoes in overground running (1) increases the anterior foot shift, (2) alters the energy contributions of hip, knee and ankle joint, and (3) reduces the lower limb muscle forces of selected hip, knee and ankle joint muscles.

## Materials and methods

### Participants

Nine male, non-professional, long-distance runners (mean ± SD: age 32.9 ± 6.1 y, height 1.78 ± 0.04 m, mass 75.7 ± 5.6 kg, leg length 0.94 ± 0.03 m) volunteered to participate in the study. All participants had previously completed the treadmill study of Wunsch et al. [7], and were familiarized in running with LEAF and FOAM footwear. All participants were heel strikers with a foot-ground angle at touch-down of at least 10 degrees, which was checked beforehand as an inclusion criterion [7]. Prior to the measurements, the participants were informed about the potential risks and discomforts and completed a written informed consent document. The study was conducted in accordance with the Declaration of Helsinki and approved by the ethics committee of the University (Approval: 13BM-12).

### General overview

After a 10-min individual warm-up, the participants ran on a 40-m indoor track with a force plate imbedded at 30-m of the runway, using both LEAF (size US 9) and FOAM (size US 9) in randomized order. Both shoes featured similar geometrical characteristics and had a mass of 327 g (LEAF) and 338 g (FOAM). To ensure consistency with previous work on treadmill running [7], a constant speed of 3 m/s was chosen. The running speed was constrained by an acoustic pacemaker (signal every 5 m) and was controlled by photocells positioned 2.5 m before and 2.5 m after the center of the force platform. The participants completed four trials with each shoe, ensuring a full foot contact on the force platform and maintaining a speed range of 3.0 ± 0.2 m/s. A period of 5 min between the shoe conditions was provided for changing the shoes.

### Instruments and calculations

Reflective markers (diameter of 15 mm) were attached to the participants according to the Cleveland Clinic Marker set (Motion Analysis Corp, Santa Rosa, USA). Kinematic and kinetic data were collected simultaneously by an eight camera three-dimensional motion analysis system (200 Hz, Vicon MX 1.3, Oxford Metrics Ltd, UK) and a force plate (1000 Hz, AMTI, Advanced Mechanical Technology Inc., Watertown, Massachusetts, USA). Kinematic and kinetic data were processed using Vicon Nexus 1.7.1 software (Vicon, Oxford Metrics Ltd, UK). Further calculations were completed using MATLAB (R2013a) and an inverse dynamic musculoskeletal modelling software (AnyBody 6.0, AnyBody Technology A/S, Aalborg, Denmark).

Raw data were filtered using a sixth order zero-lag Butterworth low pass filter with a cut-off frequency of 12 Hz for the kinematic data and 50 Hz for the kinetic data [17]. The time events of ‘heel-strike’ (HS) and ‘toe off’ (TO) used to define the stance phase were determined by a 10-N threshold applied to the vertical ground reaction force [17]. ‘Heel off’ (HO) was defined as the event when the vertical velocity of the heel marker changed from negative to positive [7].

The anterior shift of the foot was calculated as the displacement of the heel marker in the anterior-posterior direction from HS to HO [7]. Joint moments, joint angles and muscle forces were calculated using the musculoskeletal model (AMMR 1.6.2, MoCapModel) available in the AnyBody Modeling System. This model includes the Twente Lower Extremity Model (TLEM), which is based on the morphological data set for the lower extremities by Klein Horsmann et al. [18]. The data set was used to model mass, moments of inertia, and muscle sites/geometry for all segments. The model consisted of 11 body segments: head, trunk, pelvis, right and left femur, patella, tibia, talus, and foot. Each leg contained 55 muscles and mechanical effects were modelled by 159 simple muscle slips [18]. The model was scaled to match each participant’s anthropometry [19]. Inverse dynamics were performed and a third order polynomial muscle recruitment criterion was used [10, 11].

Joint power (P_j_) for the joint j (hip, knee and ankle joint) was calculated using the equation:

$$P_{j}=M_{j}∙\omega_{j}$$ (1)

with M_j_ as internal joint moment and ω_j_ as joint angular velocity retrieved from the model.

The amount of generated and absorbed energy at the hip, knee and ankle joint was calculated using trapezoidal numerical integration of the power-time curve. For each trial, the absolute values of all power and energy data were normalized to body mass.

Based on the model, lower limb muscle forces during stance phase were determined for: m. gluteus maximus (GM), m. biceps femoris (BF), m. rectus femoris (RF), m. vastus medialis (VM), m. vastus lateralis (VL), m. gastrocnemius medialis (GM), m. gastrocnemius lateralis (GL), m. soleus (SO) and m. tibialis anterior (TA). For each trial, all muscle forces were normalized to body weight (BW).

### Model validity and influence of shoe marker placement

The TLEM model was validated during walking and showed a good agreement between the estimated muscle forces and measured EMG data [20]. Furthermore, the TLEM model was previously used to analyze sprint running [12]. No studies were found using this model for analyzing midsole designs in running shoes. The calculated time courses of hip, knee and ankle power in this study, however, corresponded well with those from previous studies [21, 22] and the analyzed lower limb muscle forces showed that almost all muscles acted similarly to patterns reported the literature [11, 23].

Except the shoe marker (Fig 1) no marker were replaced between the shoe conditions. While both shoes featured similar geometrical characteristics and specific care was taken to place the markers at the identical position, a perfect match cannot be guaranteed. Therefore, a sensitivity analysis on marker placement was conducted. For one typical trial on one participant, the shoe markers were mathematically repositioned within a range of 7 mm, which represents alterations clearly above the assumed error of marker displacement by the investigator. To determine the effect of these repositions on joint energy and muscles forces the modified trials were compared with the original trial. The differences calculated for each joint were: 1) energy absorption: < 3% at the hip, < 1% at the knee and < 1% at the ankle joint; 2) energy generation: < 3% at the hip, < 1% at the knee and < 3% at the ankle joint; 3) mean muscle forces: < 1% for GM, < 3% for BF, < 2% for RF, < 1% for VM, < 1% for VL, < 2% for GM, < 2% for GL, < 1% for SO and < 4% for TA.

### Statistics

The course of the joint power data and muscle forces of all four trials for each participant and shoe condition were time normalized over the entire stance phase. Ensemble mean curves were calculated for each participant and shoe condition. Additionally, group means along the stance phase were calculated and presented as mean ± standard error curve.

For analysis, mean (± standard error) values were reported for: anterior foot shift, peak power absorption/generation and energy absorption/generation at the hip, knee and ankle joint, average muscle force during stance for each analyzed muscle. For each variable normal distribution was confirmed by the Shapiro Wilk test. A paired sample t-test was applied for analyzing a shoe difference for the variable anterior foot shift. With respect to the hypotheses of this study, for the remaining variables functional groups were built, more specifically ‘hip joint power and energy’ (G1), ‘knee joint power and energy’ (G2), ‘ankle joint power and energy’ (G3), ‘hip joint muscles’ including GM, BF, RF (G4), ‘knee joint muscles’ including RF, VM, VL, GM, GL (G5) and ‘ankle joint muscles’ including GM, GL, SO, TA (G6). For each of these six groups (G1-G6), separate MANOVAs were calculated. In case of significance, a univariate ANOVA was performed. The level of significance for the univariate tests was Bonferroni-adjusted according to the number of variables in each group. Cohen’s d_z_ was used to describe the effect size and practical relevance of differences. Effect sizes for each comparison were described as: small for d_z_ between 0.20 and 0.49, medium for d_z_ between 0.50 and 0.79 and large for d_z_ > 0.80 [24]. Level of significance was set at α < 0.05. The Statistical Package for the Social Sciences (Version 24.0; SPSS Inc., Chicago, IL, USA) was used.

## Results

The anterior shift of the foot was increased by 8 ± 1 mm for LEAF compared to FOAM (p < 0.001, d_z_ = 2.36) (Table 1 and S1 File).

**Table 1:** 

	LEAF	FOAM	diff (LEAF-FOAM)	P	d_z_
Anterior foot shift [mm]	20 ± 1	12 ± 1	8 ± 1	<0.001	2.36
*Hip joint (G1)*			*Adjusted level of significance*: *α < 0*.*0125*		
Peak power absorption [W/kg]	3.25 ± 0.45	4.28 ± 0.45	-1.03 ± 0.14	<0.001*	2.50
Peak power generation [W/kg]	2.01 ± 0.29	2.06 ± 0.42	-0.05 ± 0.23	0.853	0.06
Energy absorption [J/kg]	0.33 ± 0.02	0.37 ± 0.02	-0.04 ± 0.01	0.010*	1.11
Energy generation [J/kg]	0.10 ± 0.02	0.11 ± 0.03	-0.01 ± 0.01	0.500	0.24
*Knee joint (G2; MANOVA not sig*.*)*					
Peak power absorption [W/kg]	13.68 ± 0.65	13.36 ± 0.81	0.32 ± 0.22		
Peak power generation [W/kg]	7.87 ± 0.61	8.13 ± 0.70	-0.26 ± 0.16		
Energy absorption [J/kg]	0.62 ± 0.03	0.61 ± 0.04	0.01 ± 0.01		
Energy generation [J/kg]	0.48 ± 0.04	0.50 ± 0.05	-0.02 ± 0.01		
*Ankle joint (G3)*			*Adjusted level of significance*: *α < 0*.*0125*		
Peak power absorption [W/kg]	9.40 ± 0.38	9.76 ± 0.51	-0.36 ± 0.38	0.364	0.32
Peak power generation [W/kg]	13.08 ± 0.66	15.27 ± 0.81	-2.19 ± 0.35	<0.001*	2.08
Energy absorption [J/kg]	0.56 ± 0.02	0.57 ± 0.03	-0.01 ± 0.02	0.581	0.19
Energy generation [J/kg]	0.71 ± 0.03	0.81 ± 0.03	-0.10 ± 0.01	<0.001*	4.29
*Mean muscle force during stance*					
*Hip joint muscles (G4)*			*Adjusted level of significance*: *α < 0*.*0125*		
M. gluteus maximus [× BW]	0.18 ± 0.01	0.19 ± 0.01	-0.01 ± 0.00	0.248	0.41
M. biceps femoris [× BW]	0.25 ± 0.02	0.28 ± 0.02	-0.03 ± 0.01	0.031^t^	0.87
M. rectus femoris [× BW]	0.43 ± 0.03	0.43 ± 0.04	0.00 ± 0.01	0.953	0.02
*Knee joint muscles (G5)*			*Adjusted level of significance*: *α < 0*.*010*		
M. rectus femoris [× BW]	0.43 ± 0.03	0.43 ± 0.04	0.00 ± 0.01	0.953	0.02
M. vastus medialis [× BW]	0.59 ± 0.04	0.61 ± 0.05	-0.02 ± 0.01	0.090	0.64
M. vastus lateralis [× BW]	1.28 ± 0.09	1.32 ± 0.10	-0.04 ± 0.02	0.085	0.66
M. gastrocnemius medialis [× BW]	0.61 ± 0.04	0.67 ± 0.04	-0.06 ± 0.02	0.009*	1.15
M. gastrocnemius lateralis [× BW]	0.24 ± 0.01	0.28 ± 0.01	-0.04 ± 0.01	<0.001*	2.16
*Ankle joint muscles (G6)*			*Adjusted level of significance*: *α < 0*.*0125*		
M. gastrocnemius medialis [× BW]	0.61 ± 0.04	0.67 ± 0.04	-0.06 ± 0.02	0.009*	1.15
M. gastrocnemius lateralis [× BW]	0.24 ± 0.01	0.28 ± 0.01	-0.04 ± 0.01	<0.001*	2.16
M. soleus [× BW]	2.79 ± 0.11	3.01 ± 0.13	-0.22 ± 0.04	0.001*	1.69
M. tibialis anterior [× BW]	0.11 ± 0.01	0.11 ± 0.01	0.00 ± 0.00	0.242	0.42

* significant difference

^t^ trend

All of the calculated MANOVAs, except for G2, revealed significant multivariate main effects for the depended variable shoe (G1: Wilks’ λ = 0.033, F = 36.89, p = 0.001, G2: Wilks’ λ = 0.419, F = 1.74, p = 0.278, G3: Wilks’ λ = 0.031, F = 38.94, p = 0.001, G4: Wilks’ λ = 0.224, F = 6.94, p = 0.022, G5: Wilks’ λ = 0.028, F = 17.61, p = 0.019, G6: Wilks’ λ = 0.025, F = 48.23, p < 0.001). Detailed information is presented in Table 1.

**Fig 2:**
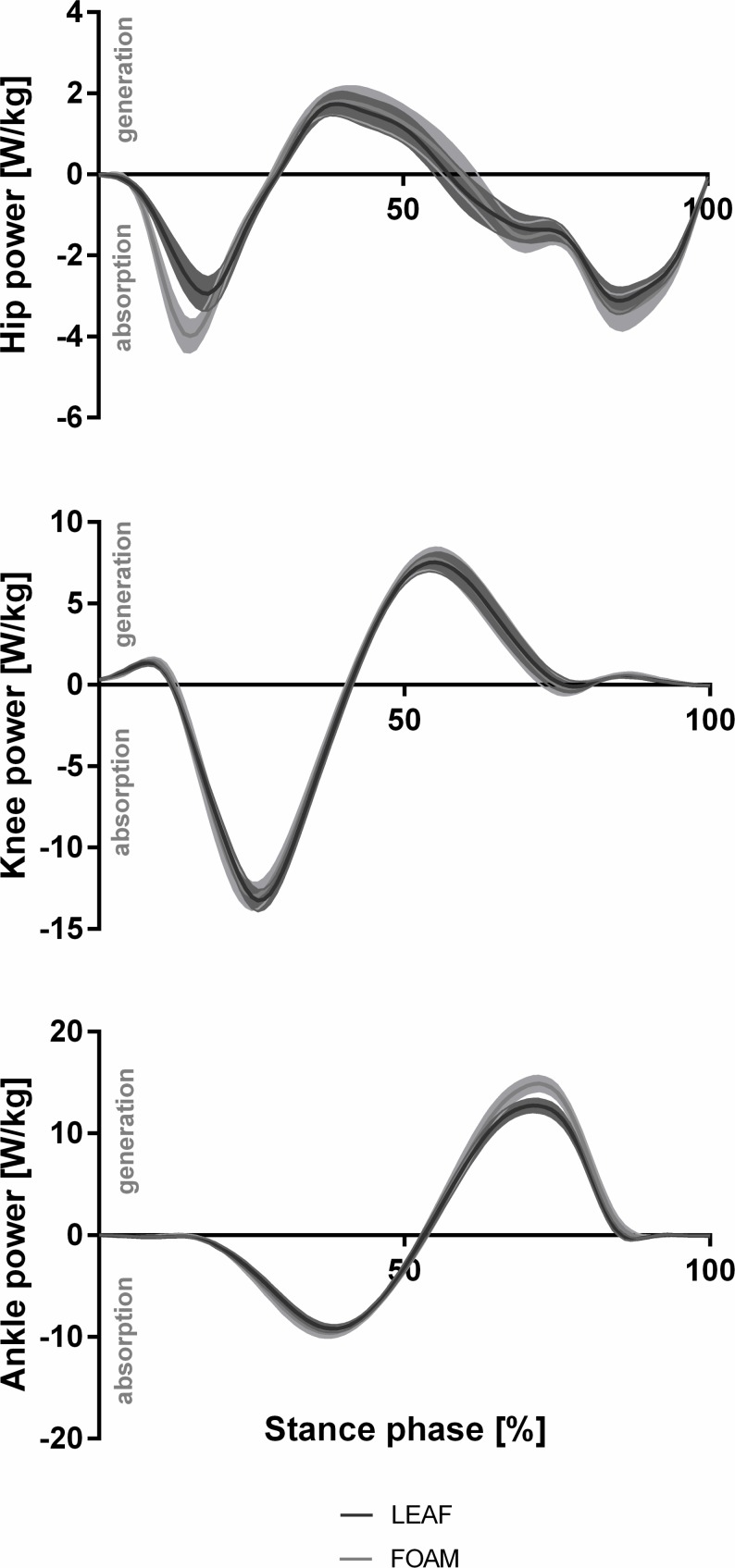
Joint power. Joint power trajectories during stance (mean ± standard error) for the hip, knee and ankle joint in running with LEAF and FOAM shoes.

The time profiles of the joint power for the hip, knee and ankle joint are presented in Fig 2. The differences between LEAF and FOAM occurred at the hip joint primarily during the braking phase and at the ankle joint predominantly during the push-off phase. At the hip, a reduction of 32% in the peak power absorption (p < 0.001, d_z_ = 2.50) and 11% in the energy absorption (p = 0.010, d_z_ = 1.11) was found for LEAF compared with FOAM, whereas at the ankle joint, a reduction of 17% in peak power generation (p < 0.001, d_z_ = 2.08) and 13% in the energy generation (p < 0.001, d_z_ = 4.29) occurred (Table 1 and S1 File).

**Fig 3:**
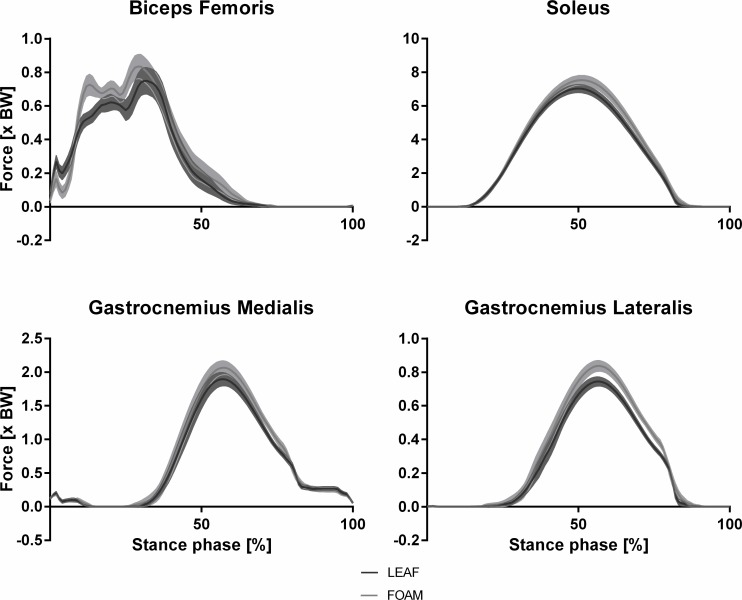
Muscle forces. Muscle force trajectories during stance (mean ± standard error) for m. biceps femoris (BF), m. gastrocnemius medialis (GM), m. gastrocnemius lateralis (GL) and m. soleus (SO).

The trajectories of the muscle forces during stance phase of BF, GL, GM and SO are presented in Fig 3 indicating that both average and peak muscle forces were affected by the midsole design. The average muscle forces (Table 1) revealed a reduction for LEAF compared to FOAM in GL (15%, p < 0.001, d_z_ = 2.16), GM (9%, p = 0.009, d_z_ = 1.15), SO (8%, p = 0.001, d_z_ = 1.69) and a trend towards reduction was found for BF (12%, p = 0.031, d_z_ = 0.87).

## Discussion

This study investigated the effects of LEAF compared to FOAM shoes on anterior foot shift, joint energy and lower limb muscle forces in overground running at a constant speed of 3 m/s. The main findings were that the participants responded to the structured midsole design showing (1) an increased anterior foot shift (hypothesis accepted), (2) a reduced energy absorption at the hip and energy generation at the ankle joint (hypothesis partly rejected) and (3) reduced lower limb muscle forces, particularly for three muscles around the ankle joint (GL, GM, SO) and a trend toward a reduction for BF (hypothesis partly rejected).

The increase in anterior foot shift in running with LEAF compared to FOAM was similar to treadmill running (8 ± 1 mm [7]). This shows that, on the one hand, the participants responded to LEAF in overground running similarly as in treadmill running. On the other hand, this indicates that the mechanical behavior of the midsole deformation during ground contact in treadmill and in overground running are similar.

The effect of this response on the energy demand in running was determined within a first step using a joint level approach. This approach was used to identify the sources and magnitudes of mechanical joint power and the contribution of energy absorption and generation to the total energy needed for performing the running task [9, 21]. Differences between LEAF and FOAM were found at the hip and the ankle joint. While running with LEAF, the reduction of hip joint energy was primarily found in the first half of the braking phase, the reduction of the ankle joint energy occurred in the second half of stance indicating that also the push-off phase was affected by the midsole design (Fig 2). As hypothesized, the reduced energy loss during the braking phase seemed to be derived from the rearfoot leaf springs leading to an anterior shift by 8 mm and a reduction of the horizontal braking force on the centre of mass [7, 25]. During the push-off phase, the short forefoot leaf springs contact ground and no additional anterior foot shift can be observed [7]. Thus, the reduced energy loss during the push-off phase seems to be also related to the energy saving during the braking phase. It can be concluded from the reduced horizontal braking of the centre of mass that less energy is needed during the push-off phase to accelerate the centre of mass for maintaining the constant running speed. Therefore, the midsole design of LEAF appeared to successfully exploit the concept of minimizing energy loss during running [1, 2].

The differences in lower limb muscle forces between LEAF and FOAM indicate which muscles were adjusted by the locomotor system in response to the midsole design for generating the movement output. Significant reductions of muscle forces comparing LEAF and FOAM occurred in GL (15%), GM (9%) and SO (8%), which accounted for the reduced energy generation at the ankle joint. Furthermore, a trend towards a reduction of lower limb muscle forces between LEAF and FOAM was found in BF (12%), explaining the reduced energy absorption at the hip joint. Previous studies showed that participants adapted the activity of lower limb muscles in response to the midsole stiffness and to varying midsole wedges [26, 27]. Based on the current study, it can be concluded that also structural changes of the midsole have the potential to affect the lower limb muscle mechanics.

In general, muscles consume metabolic energy to generate joint energy [15]. Thus, the observed reductions and trends in muscle forces can affect the total metabolic energy expenditure in running with LEAF compared to FOAM. According to Kyrolainen et al. [16] the BF provides the greatest impact on economy and is one of the largest extensor muscles at the hip, which consumes a considerable amount of metabolic energy when active due to its high muscle mass [22, 28]. Therefore, it is favorable to conserve metabolic energy by reducing the hip extensor muscle activity [22], which was demonstrated in this study by the trend towards reductions of muscle forces.

The muscles contributing primarily to the horizontal braking and propulsion of the center of mass during stance have a substantial impact on running economy [16]. During the braking phase, the quadriceps muscle group is the largest contributor to deceleration of the horizontal motion of the center of mass [21, 29] and during the propulsion phase the triceps surae muscle group is the greatest contributor to forward acceleration of the centre of mass [23, 29]. In running with LEAF compared to FOAM shoes, RF, VL and VM showed no differences in muscle forces during the braking phase. The muscle force of the triceps surae group (GL, GM and SO; Fig 3), however, was reduced by 11% when running with LEAF compared with FOAM. Consequently, this group produced lower muscle forces for propulsion in running at constant speed.

For the same participants with the same test shoes (LEAF and FOAM) at the same running speed (3 m/s), a reduction of oxygen consumption of 2% with LEAF shoe was found in treadmill running [7]. Hence, the positive effect of LEAF on oxygen consumption in this study can be at least partly explained by the observed reductions of the lower limb muscle forces combined with the reductions of energy absorption and generation at the hip and ankle joint. This is supported by the consideration that for marathon running, a total physiological energy consumption during one foot contact is estimated to be 6.61 J/kg [1]. The sum of measured reductions for LEAF compared to FOAM shoes in absorbed energy at the hip (0.04 J/kg) and generated energy at the ankle (0.11 J/kg) was 0.15 J/kg. This represents 2% of the total energy required and presents a similar reduction of oxygen consumption [7]. It has to be noted, however, that further relevant energy forms, e.g. thermodynamic, chemical, etc. [1] were not taken into account.

Finally, the gained results could provide an important background concerning injury prevention. Muscles have been shown to be the major contributors to the joint contact forces [30–32]. Thus, reduced muscle forces could lead to reduced joint load. Sinclair [33] has shown that the peak Achilles tendon force was significantly reduced using LEAF compared to conventional footwear. These results are in line with the reduced triceps surae muscle forces found in the current study.

One limitation of this study was the small sample size. It should be noted, however, that the presented results are well in line with previous studies analyzing the same test shoes [7, 34]. Thus, it can be concluded that for runners responding to LEAF the results found in this study are plausible. The second limitation was the slightly reduced shoe mass of 11 g for LEAF compared to FOAM. This explains approximately 0.11% of the differences in oxygen consumption [35] and may also contribute to a small extent to the observed changes in joint mechanics and muscle forces. Furthermore, this and the previous studies comparing LEAF with FOAM shoes only investigated the midsole effects in non-fatigued running. From a practical perspective it would be interesting to examine the joint power joint energy and leg muscle forces under fatigued conditions [36].

## Conclusion

Knowledge of the energy generation and absorption of the lower limb joints and the biomechanical function of the lower limb muscles is important for improving the understanding of performance in running with different running shoes. Hereby musculoskeletal models could provide valuable information for the sport shoe research. This study showed that structural changes of the midsole have the potential to affect joint energy and lower limb muscle forces in running. Furthermore, these findings are consistent with previous studies indicating that running with LEAF compared to FOAM enhances running economy [7]. Thus, the structured midsole shoe seems to be suitable for heel strike runners and may enhance running performance by reducing lower extremity joint energy due to force reductions in lower extremity muscles.

## Supporting information

S1 File Kinematics and kinetics. (XLS)

S2 File Muscle forces. (XLS)

S3 File Wunsch-2016-ConferenceAbstractFBG. (PDF)
