# Supplementary material for: Effects of a leaf spring structured midsole on joint mechanics and lower limb muscle forces in running
Source: PLoS One. 2017 Feb 24;12(2):e0172287. doi: 10.1371/journal.pone.0172287 (PMC5325235; doi:10.1371/journal.pone.0172287)
Supplement: S3 File — (PDF) [file pone.0172287.s003.pdf]

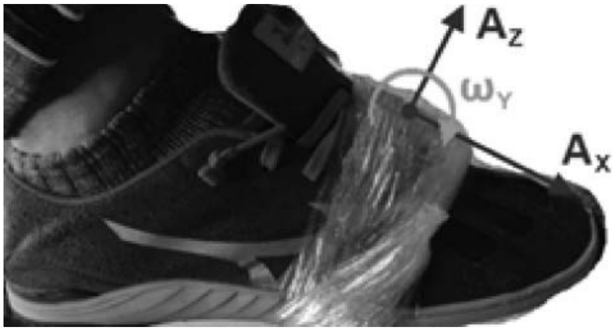

Figure 1. A shoe with the sensor, wrapped in food wrap film, attached to it. The arrows represent the directions in which the signals are measured. The missing arrows (ω_x, ω_z and A_y) can be derived using the right-hand rule for axis orientation.

sample can be drawn from a confidence region, giving preference to the higher probability. To ensure this algorithm has an accuracy that is in line with recent laboratory studies (around 95% or higher), the algorithm is tested without limiting the radius of the confidence region to a certain number of standard deviations. This ensures that all testing data are classified as one of the four activities.

Results

The classification accuracy of the algorithm is depicted in Table 1. It was found that walking, running and jumping were classified with an accuracy of over 98%. Jogging was classified less accurately, but with an accuracy of 95.9% it was still in line with results of recent studies.

Table 1. Confusion matrix for the model. The values in the columns without unit represent the number of 1 second windows that were classified.

	Walk	Jog	Run	Jump	Total	Accuracy (%)
Walk	1751	2	0	16	1769	99.0
Jog	8	757	3	21	789	95.9
Run	0	0	212	0	212	100
Jump	0	4	0	215	219	98.2

Discussion and conclusion

The current method has an accuracy that is in line with those obtained in recent studies (e.g. Preece et al., 2009), meaning a classification accuracy of over 95%. We found that jogging is the most erroneous classified activity, while running is the most accurately classified activity. It should be noted that in real-life situations, the accuracies are very likely to be lower due to disturbances, different sensor alignment, etc.

In this study, the size of the confidence region was chosen large enough to cover all samples of testing data. Therefore, all the data were classified as belonging to one of the activities, as are usual for laboratory studies. When smaller confidence regions are chosen, they will not cover all the testing data samples, resulting in some samples not being classified as activity. The choice of the size of the confidence regions has a big influence on the number of samples that are classified correctly.

Disclosure statement

No potential conflict of interest was reported by the authors.

References

Chang, S. *et al.* (2013). *ISB 2013 Brazil*.
IHS iSuppli Research (2013). *IHS Online Pressroom*.
Preece, S. *et al.* (2009). *IEEE Biomedical EngIneering, 56*, 871–879.

Downloaded by [Universitat Salzburg] at 01:04 05 August 2016

Effects of a leaf spring structured midsole on lower limb muscle forces in running

Tobias Wunsch*, Nathalie Alexander, Josef Kröll and Hermann Schwameder

Department of Sport Science and Kinesiology, University of Salzburg, Hallein-Rif, Austria

(*Received 6 February 2015; accepted 25 March 2015*)

Introduction

The major goal of lower leg muscles during ground contact in running is to produce joint torques for executing the movement task. Furthermore, muscles are activated to minimise the vibrations of soft-tissue compartments based on a specific input signal (Nigg & Wakeling, 2001).

*Corresponding author. Email: tobias.wunsch@sbg.ac.at

http://dx.doi.org/10.1080/19424280.2015.1039082

Running shoes and more specifically the midsole material can change the input signal leading to an altered muscle activation of the leg muscles (Nigg, Stefanyshyn, Cole, Stergiou, & Miller, 2003). It remains unclear, if next to the midsole material also, structural changes of the midsole can substantially influence the lower limb muscle activity. These changes, however, in muscle activity could affect the work done by the human locomotor system and, therefore, could influence running economy (Nigg et al., 2003).

Purpose of the study

The purpose of this study was to investigate the effects of a leaf spring structured midsole on lower leg muscle forces in heel–toe running using a musculoskeletal model.

Methods

Eight male heel-strikers (age: 34 ± 3 yrs, height: 1.79 ± 0.01 m, mass: 73.6 ± 2.4 kg, training: >20 km/week) participated in the study. Two pairs of shoes were tested: a leaf spring structured midsole shoe (LEAF; 327 g) and a reference shoe consisting of a standard foam EVA midsole (FOAM; 338 g).

The participants ran on a 40-m indoor track with an embedded force plate located at 30 m of the runway with a running speed of 3 ± 0.2 m/s. The participants completed four valid trails with contact on the force platform (two left, two right) and within the prescribed velocity range.

Reflective markers were attached to the participants according to the Cleveland Clinical Marker set. Kinematic and kinetic data were collected with an eight-camera motion analysis system (Vicon, 200 Hz) and the force plate (AMTI, 1000 Hz). Processed kinematic and kinetic data were imported into an inverse dynamic musculoskeletal modelling software (AnyBody). The major leg muscle forces – gluteus maximus (GL), rectus femoris (RF), biceps femoris caput longum (BF), vastus medialis (VM), vastus lateralis (VL), gastrocnemius (GA), soleus (SO), tibialis anterior (TA) – were calculated using the musculoskeletal model (AMMR 1.6.2, MoCapModel).

Group differences for the mean force during stance of each muscle and participant were statistically tested using paired t-tests ($p < 0.05$). Cohen's d_z described the relevance of differences.

Results

The mean muscle forces (Figure 1) show a significant reduction with LEAF compared to FOAM for VL (4%), VM (4%), GA (9%), and SO (7%). The other muscles revealed no differences between the midsole conditions. Exemplarily,Figure 2 shows the time courses of GA and SO muscle forces during stance.

Discussion and conclusion

The results of this study indicate that in running at a constant velocity with LEAF compared to FOAM, the locomotor system requires less muscle force of VL, VM, GA, and SO to generate the movement output and to minimise soft-tissue vibrations. Also, Wakeling, Pascal and Nigg (2002) and O'Connor, Price and Hamill (2006) reported

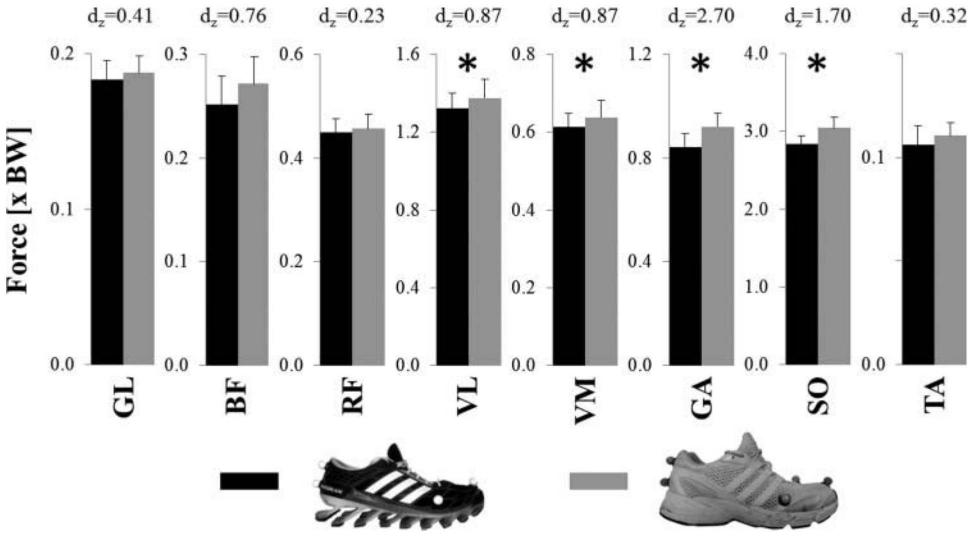

Figure 1. Mean ± SE muscle force for LEAF (black) and FOAM (grey).

Downloaded by [Universitat Salzburg] at 01:04 05 August 2016

Downloaded by [Universitat Salzburg] at 01:04 05 August 2016

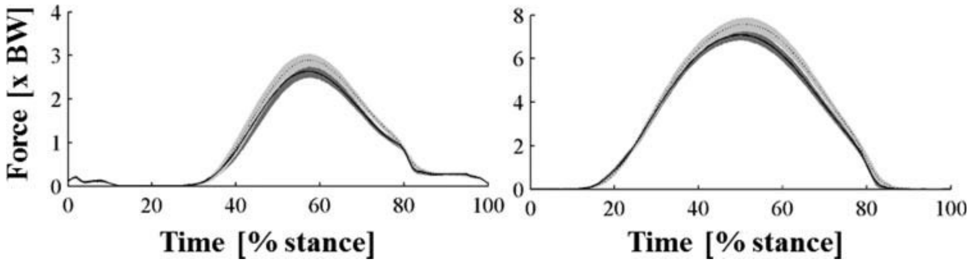

Figure 2. Mean ± SE GA (left) and SO (right) muscle force for LEAF (solid black) and FOAM (dashed grey).

that participants adjust lower leg muscle activity in response to the midsole stiffness and to different midsole wedges. Based on the tested shoes in this study, it can further be concluded that next to the midsole material also, structural changes of the midsole have the potential to affect lower limb muscle forces.

It remains unclear, if the observed reductions in VL, VM, GA, and SO muscle forces have direct implications on running economy (Nigg et al., 2003). In treadmill running, however, the same participants showed a reduced oxygen consumption with LEAF compared to FOAM (2%; Wunsch et al., n.d.). Additionally, according to Kyrolainen, Belli and Komi (2001), the BF showing a trend towards a reduction in muscle force (8%, $d_z = 0.76$) with LEAF seems to have the greatest impact on economy. Thus, it could be speculated that the observed changes in muscle forces in response to the structured midsole concept are responsible for the improvements in running economy.

One limitation of the study was the slightly varying shoe mass by 11 g. This explains approximately 0.11% (Frederick, 1986) of the $\dot{V}O_2$ differences and may also contribute to a small extend to the observed changes in muscle activity.

Disclosure statement

No potential conflict of interest was reported by the authors.

References

Frederick, E.C. (1986). Kinematically mediated effects of sport shoe design: A review. *Journal of Sports Science, 4*(3), 169−184.

Kyrolainen, H., Belli, A., & Komi, P.V. (2001). Biomechanical factors affecting running economy. *Medicine and Science in Sports and Exercise, 33*(8), 1330−1337.

Nigg, B.M., & Wakeling, J.M. (2001). Impact forces and muscle tuning: A new paradigm. *Exercise and Sport Science, 29*(1), 37−41.

Nigg, B.M., Stefanyshyn, D., Cole, G., Stergiou, P., & Miller, J. (2003). The effect of material characteristics of shoe soles on muscle activation and energy aspects during running. *Journal of Biomechanics, 36*(4), 569−575.

O'Connor, K .M., Price, T .B., & Hamill, J. (2006). Examination of extrinsic foot muscles during running using mfMRI and EMG. *Journal of Electromyography and Kinesiology, 16*(5), 522−530.

Wakeling, J.M., Pascal, S.A., & Nigg, B.M. (2002). Altering muscle activity in the lower extremities by running with different shoes. *Medicine & Science in Sports & Exercise's, 34* (9), 1529−1532.

Wunsch, T. *et al.* (n.d.). Under review.

Shoe surface traction and impact tester

Poovan Pandiyar Ganason [a,b]* and Thuvendran Pandiyar Ganason[c]

[a]*National Sports Institute, Research & Technology, Kompleks Sukan Negara, Kuala Lumpur, Malaysia;* [b]*Research & Technology, Ipoh, Malaysia;* [c]*GMP Genesis Enterprise, Research and Technology, Ipoh, Malaysia*

(*Received 6 February 2015; accepted 25 March 2015*)

Introduction

Speaking of constructing a shoe, the measurement of traction on shoe-ground interface and its impact on shoe bed is very much crucial for optimization in sports performance and mitigating risk of injury to the wearer. In such, voluminous experimental methods have been created to measure translational and rotational friction of footwear and the surface whereby, carefully designed mechanical devices are preferred over human subject in laboratory tests due to its low variability (Frederick, 1993). However, the methodologies used by these mechanical devices vary greatly in traction extraction procedures, loading

*Corresponding author. Email: poovanganason@gmail.com

http://dx.doi.org/10.1080/19424280.2015.1039083
